# Real-World Insights into Stage I–III Non-Small Cell Lung Cancer in Spain in the Pre-Immunotherapy Era Using AI Techniques: The IntellyLUNG Study

**DOI:** 10.3390/life16071119

**Published:** 2026-07-05

**Authors:** Jesús Corral Jaime, Javier de Castro, Aitor Azkarate, Gema García Ledo, Antonio Calles, Raquel Marsé, Ana Sofia de Freitas Matos Parreira, Julia Villamayor, Laura Gutiérrez-Sainz, Javier-David Benítez-Fuentes, Diego Casado Elía, Natalia Gutiérrez, Marta Arregui Valles, Eduard Sarró, Noelia López, Savana Research Group

**Affiliations:** 1 Hospital Universitario de Jerez, 11407 Cádiz, Spain; 2 Hospital Universitario La Paz, Instituto de Investigación Sanitaria del Hospital Universitario La Paz (IdiPAZ), Universidad Autónoma de Madrid (UAM), 28046 Madrid, Spain; 3 Hospital Universitario Son Espases, 07120 Palma de Mallorca, Spain; 4 Hospital Universitario HM Sanchinarro, HM Hospitales, Centro Integral Oncológico Clara Campal (HM-CIOCC), 28050 Madrid, Spain; 5 Instituto de Investigación Sanitaria HM Hospitales, 28050 Madrid, Spain; 6 Hospital General Universitario Gregorio Marañón, 28007 Madrid, Spain; 7 Savana Research S.L., 28004 Madrid, Spain; 8 Medical Affairs Department, Merck Sharp & Dohme (MSD) Spain, 28027 Madrid, Spain

**Keywords:** NSCLC, natural language processing, electronic health records, health resources, Spain

## Abstract

Treatment of non-small cell lung cancer (NSCLC) has been transformed by immunotherapy and targeted therapies. We aimed to characterize clinical features, treatment patterns, and healthcare resource use in patients with early and locally advanced NSCLC before incorporation of these therapies. This retrospective observational study included adults diagnosed with stage I–III NSCLC at four Spanish hospitals between 2014 and 2018, with follow-up until 2021, using artificial intelligence to extract data from electronic health records. A total of 951 patients were included (34.7% stage I, 16.7% stage II, 48.6% stage III), with a median age of 66 years and 31.9% female. Surgery was performed in 78.5% of stage I, 74.8% of stage II, and 35.5% of stage III patients. Among surgical patients, 62.5% received adjuvant chemo- and/or radiotherapy, 20.8% neoadjuvant therapy, and 15.7% both; among non-surgical patients, chemoradiotherapy was the most common treatment (50.4%). Beyond hospitalization, outpatient visits were the most frequently used healthcare resource. These findings provide a historical benchmark of NSCLC care before introduction of immunotherapy and targeted therapies in these settings, highlighting treatment variability and the need for earlier diagnosis, structured treatment pathways, and multidisciplinary management.

## 1. Introduction

In Spain, an estimated 34.,908 new cases of lung cancer will be diagnosed in 2026, with 66% of these occurring in men [1]. While incidence in men has decreased in recent years, rates in women have increased, with an estimated incidence in 2026 that is 2.37 times higher than for 2007 [1]. Non-small cell lung cancer (NSCLC) accounts for 80–90% of all lung cancer cases [2]. Early stage (I-II) and locally advanced (III) disease represent a critical window for providing treatment with curative intent, although 5-year survival rates in stage III disease have been low, ranging between 12% and 41% [3]. For early-stage disease, surgery is the foundation of treatment, with lobectomy recommended in most cases [4]. Historically, adjuvant chemotherapy after surgery was recommended to improve survival in patients with stage II–III tumors, while post-surgery radiotherapy (before chemotherapy) was considered for those with incomplete resection [5]. In unresectable stage III disease, the standard approach was concurrent chemoradiotherapy, with sequential chemo- and radiotherapy when concurrent administration was not possible [4].

The arrival of targeted therapies and immunotherapy has dramatically changed the treatment landscape for both resectable and unresectable NSCLC. For patients with tumors harboring targetable oncogenic alterations, current guidelines recommend targeted therapies, and these patients are generally not considered candidates for immune checkpoint inhibitors [6]. In this context, adjuvant osimertinib is now recommended after complete tumor resection in stage IB-IIIA patients with epidermal growth factor receptor (EGFR) mutation-positive tumors [5]. In unresectable EGFR-mutated disease, recent guideline updates now recommend consolidation therapy with osimertinib, with investigation in this area evolving rapidly [7,8]. In resectable NSCLC without actionable genomic alterations, immunotherapy has been integrated into neoadjuvant, perioperative, and adjuvant settings using immune checkpoint inhibitors [9]. In the neoadjuvant setting, nivolumab plus chemotherapy has demonstrated improved event-free survival and overall survival compared with chemotherapy alone [10]. In perioperative approaches, nivolumab, pembrolizumab, and durvalumab have shown improvements in event-free survival and pathological response rates compared with chemotherapy alone, with pembrolizumab also showing significant overall survival benefit [11,12,13,14,15]. In the adjuvant setting, atezolizumab demonstrated disease-free survival benefit and was approved by the European Medicines Agency for patients whose tumors express programmed death ligand (PD-L1) equal to or more than 50%, while pembrolizumab has demonstrated a significant disease-free survival benefit and was approved regardless of tumor PD-L1 expression status in resectable patients after an adjuvant platinum-based chemotherapy [16,17]. In unresectable stage III disease, consolidation therapy with durvalumab is now the standard of care for patients without progression after chemoradiotherapy with curative intent [5].

These advances demand multidisciplinary management strategies, expanding the range of therapeutic options and clinical decision-making pathways [18]. In this context, understanding current practice requires contextualization within prior treatment paradigms. Therefore, this study aimed to describe the real-world clinical characteristics, treatment strategies, and healthcare resource use in patients with stage I, II, or III NSCLC in Spain diagnosed between 2014 and 2018 and followed until 2021. This period preceded the incorporation of immunotherapies and targeted therapies in early-stage and locally advanced disease, although these treatments were already used in advanced stages during part of the study timeframe. To this end, we used artificial intelligence (AI) techniques, including natural language processing (NLP) and machine learning (ML), to extract data from electronic health records (EHRs). This historical perspective provides a baseline for understanding current challenges and evolving priorities in NSCLC management, including early detection strategies and screening, biomarker testing, multidisciplinary approaches, structured patient pathways, and the emerging needs of long-term survivors.

## 2. Materials and Methods

### 2.1. Study Design

**Figure 1:**
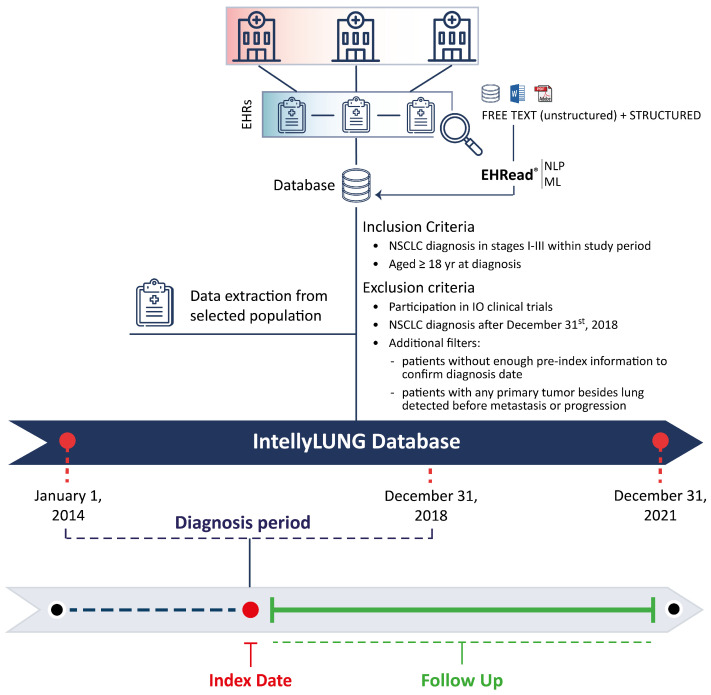
Study design. Structured and unstructured free-text information from EHRs from the 4 participating sites was organized into a study database. Inclusion and exclusion criteria and additional filters were applied to define the study population. Variables were extracted from the database at different time points and analyzed to address the study objectives. EHR, electronic health record; IO, immunotherapy; ML, machine learning; NLP, natural language processing; NSCLC, non-small cell lung cancer.

IntellyLUNG was a multicenter observational study using retrospective AI-powered data extraction to analyze adult patients with early or locally advanced NSCLC who received care at one of the participating study sites. Source data for the study included unstructured and structured information captured in EHRs of patients from four hospitals in the Spanish National Healthcare Network: Hospital Universitari Son Espases (Mallorca), Hospital Universitario La Paz (Madrid), Hospital General Universitario Gregorio Marañón (Madrid), and Grupo Hospitalario HM (Madrid). Participating centers included a combination of secondary and tertiary care hospitals, as well as a large private hospital network. Using NLP and ML, data were extracted from EHRs spanning the study period of 1 January 2014 to 31 December 2021, which included a diagnosis period from 1 January 2014 to 31 December 2018. For all patients, the date of inclusion in the study (index date) was the timepoint within the diagnosis period when NSCLC was identified earliest in their EHRs. A cross-sectional analysis of all patients was performed at their index date, and a longitudinal analysis was conducted over a follow-up period spanning from the index date (a point between January 2014 to December 2018) until their last EHR available, or the end of the study in December 2021 (Figure 1).

### 2.2. Study Population

From the total screening set provided by the participating hospitals, the source population comprised all patients with a clinical diagnosis of NSCLC during the diagnosis period, as determined from information found in EHRs and extracted using NLP. For inclusion in the study population, patients with NSCLC had to have cytologically or histologically confirmed disease of stage I, II, or III, and had to be at least 18 years of age at the time of diagnosis. Patients diagnosed outside of the diagnosis period were excluded from the study population. Patients treated with immunotherapy, either as part of expanded access programs or clinical trials, for early or locally advanced NSCLC were also excluded. After application of the inclusion and exclusion criteria, additional filters were applied to exclude patients for whom the available pre-index information was insufficient to reliably ascertain incident disease status. Specifically, these were patients with few or no clinical records preceding the index date, which limited our ability to distinguish newly diagnosed cases from patients with a prior diagnosis whose earlier disease history was not captured in the available EHR data. Patients with any primary tumor other than lung detected before metastasis or progression were also excluded to avoid misinformation related to other cancers. For analyses, the patients included in the study population were stratified according to NSCLC disease stage at diagnosis and whether or not they underwent surgery.

### 2.3. Variable Extraction from EHRs

The study variables were selected and validated by clinical experts. Demographic characteristics and healthcare resource use variables were extracted from structured EHR data, while variables related to comorbidities, clinicopathological characteristics (including histological subtype and biomarker profile), surgery type, treatments, imaging tests, and diagnostic procedures were derived from unstructured free text. For each variable assessed at a given time point, the observation nearest to that time point within the predefined reference window was considered. These reference windows were designed to accommodate variations in routine clinical practice across patients, clinicians, and healthcare institutions, thereby maximizing the availability and capture of relevant information from EHRs. Detailed definitions of the reference windows applied to each variable or category are provided in the corresponding table footnotes.

Extraction and analysis of clinical data recorded as unstructured free text in anonymized EHRs, together with their contextual information, was performed using EHRead^®^ technology, which leverages NLP and ML techniques to transform free-text data into organized, standardized outputs using SNOMED CT terminology (Medsavana S.L., Madrid, Spain) [19,20,21,22,23,24,25,26]. The source EHRs consisted of all outpatient clinical records, discharge reports, emergency reports, and other available clinical notes and records from each participating site. A predefined list of SNOMED CT terms corresponding to the study variables was developed, incorporating synonyms and acronyms commonly used in clinical practice. The system automatically detected these entities within unstructured clinical narratives, mapped them to standardized SNOMED CT concepts, and enriched them with contextual attributes, including negation and temporality. The extracted entities and their associated metadata (e.g., clinical department, document type, and contextual attributes) were subsequently integrated into a structured database, which served as the basis for deriving and analyzing the study variables. The clinical accuracy of the conceptual definitions and entity mapping was reviewed by medical research experts and oncologists specialized in NLP. The performance of EHRead^®^ in accurately extracting key study entities at report level was evaluated using previously published methods [20], with the results summarized in Table S1. All evaluated entities showed F1-scores ranging between 0.80 and 0.98, apart from “stereotactic radiotherapy”, which showed an F1-score of 0.73.

After extraction, variables were reconstructed based on their conceptual definitions by applying dedicated data-wrangling operations to their mapped entities drawing on contextual information provided by EHRead^®^’s NLP models and structured metadata. The NLP models used by EHRead^®^ included a set of general models, as well as specific models developed to address the objectives of the study (Supplementary Tables S2 and S3). Disease stage was extracted using two dedicated NLP models (Supplementary Table S3). When multiple classifications were identified, the value closest to the earliest NSCLC mention was selected if separated by >3 months; otherwise, classifications within the same 3-month period were prioritized as follows: specific stage designation, pathological TNM (pTNM), clinical TNM (cTNM), and incomplete or ambiguous TNM.

Further details regarding data source, data processing, data operations to construct relevant variables and filters, and EHRead^®^ performance are shown in the Supplementary Materials (Sections S1.1, S1.2, S1.3 and S1.4, respectively).

### 2.4. Statistical Analysis

Descriptive analyses were conducted on the total study population and stratified by disease stage and surgical treatment. Categoric variables were summarized as frequencies, presenting the number and percentage of patients. Numerical variables were summarized using mean, standard deviation, and median, interquartile range (Q1, Q3). Summary statistics were calculated based on all patients with available values. Department usage rates (visits per 100 patients per year) were calculated as the sum of all observed department visits of each resource divided by the sum of all observed follow-up times. Usage rates are presented as rates per person-year of follow-up. Since anatomic pathology records were received from only 3 of the 4 participating sites, a sensitivity analysis was performed to determine whether the missing pathology data impacted biomarker detection results (Section S1.5 of the Supplementary Materials). Due to the limited number of sites and the need to preserve data anonymity, fully disaggregated center-level analyses are not reported.

## 3. Results

### 3.1. Study Population

**Figure 2:**
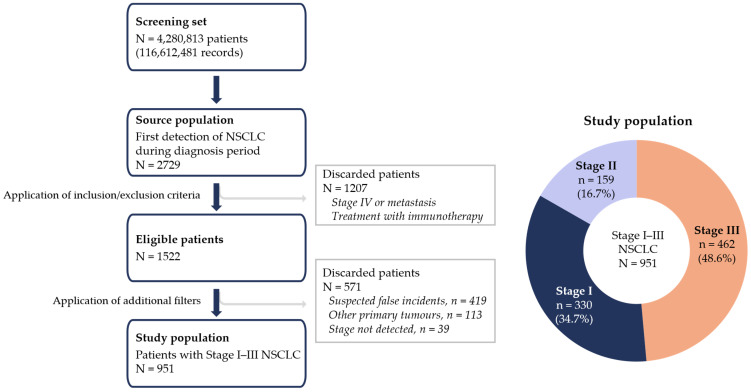
Patient flow chart. From a screening set of 4,280,813 patients, a study population of 951 adult patients with stage I, II, or III NSCLC diagnosed between 2014 and 2018 and followed up until 2021 was selected.

From a screening set of 4,280,813 patients with 116,612,481 EHRs, 951 adult patients with a known diagnosis of stage I, II, or III NSCLC were included in the study population (Figure 2). A third of the patients in the study population were diagnosed with stage I NSCLC (n = 330, 34.7%), and 159 (16.7%) with stage II. Patients diagnosed with locally advanced (stage III) disease comprised nearly half of the study population (n = 462, 48.6%, Figure 2). In all patients with stage I, II, or III NSCLC, the median (Q1, Q3) age at diagnosis was 66 years (58, 72), and 303 patients (31.9%) were female (Table 1). More than half were current smokers (n = 499, 52.5%), and 134 (14.1%) were former smokers. The most common histological subtype detected in the study population was adenocarcinoma (57.6%), followed by squamous carcinoma (27.9%). The most prevalent comorbidities identified were hypertension (50.9%), chronic obstructive pulmonary disease (42.7%), dyslipidemia (35.3%), diabetes (22.1%), and emphysema (13.7%) (Table 1). Biomarkers were largely untested in the study population (Supplementary Table S4). Of the ten biomarkers examined in the study, only EGFR, anaplastic lymphoma kinase (ALK), and PD-L1 were tested in more than 10% of patients. The most frequently tested was EGFR, which was tested in 316 patients (33.2%), of whom 79 (25.0%) had alterations. ALK was tested in 202 patients (21.2%), of whom 15 (7.4%) had alterations, and tumor proportion score (TPS) for PD-L1 expression was tested in 155 patients (16.3%), of whom 74 (47.7%) had ≥1% expression. The proportions of biomarker testing and results detected when considering only the 3 hospitals with anatomic pathology records included in the study database were similar to those of the overall study population (Supplementary Table S4).

### 3.2. Surgical and Treatment Approaches

In the study population, 542 patients had undergone thoracic surgery, including 398 (73.4%) who underwent lobectomy, and 64 (11.8%) who underwent pneumonectomy (Table 2). Surgery was performed in 259 of 330 patients with stage I (78.5%) NSCLC, 119 of 159 with stage II (74.8%), and in 164 of 462 (35.5%) with stage III. Of the patients who underwent surgery, 339 (62.5%) received adjuvant chemotherapy and/or radiotherapy treatment, 113 (20.8%) neoadjuvant treatment, and 85 (15.7%) both adjuvant and neoadjuvant treatment (Table 2). Importantly, the recorded stage may reflect downstaging following neoadjuvant treatment in those cases where pathological TNM (pTNM) was prioritized over clinical TNM (cTNM), according to the predefined staging hierarchy (Section S1.3 of Supplementary Materials). Among patients classified as stage I who received neoadjuvant therapy, 12 out of 39 had evidence of a higher earlier clinical stage, and, similarly, among those classified as stage II, 3 out of 16 had a prior stage III. Overall, these 15 patients represented 2.8% of all surgical patients (15/542) and 27.3% of stage I–II surgical patients receiving neoadjuvant therapy (15/55). The potential impact of staging reassignment on neoadjuvant therapy rates is further explored in Supplementary Results and Table S5.

A total of 409 patients in the study population did not undergo surgery (43.0%), of whom 40 (9.8%) received chemotherapy alone, 45 (11.0%) received radiotherapy alone, and 206 (50.4%) received chemoradiotherapy (Table 3). Chemoradiotherapy was detected for 175 patients with stage III disease who did not undergo surgery (58.7%), for 19 (47.5%) with stage II, and for 12 (16.9%) with stage I. Of the 206 patients who received chemoradiotherapy, treatment was administered concomitantly in 147 (71.4%), and sequentially in 51 (24.8%). For 118 patients who did not have surgery (28.9%), no active treatment was detected.

### 3.3. Healthcare Resource Use

**Figure 3:**
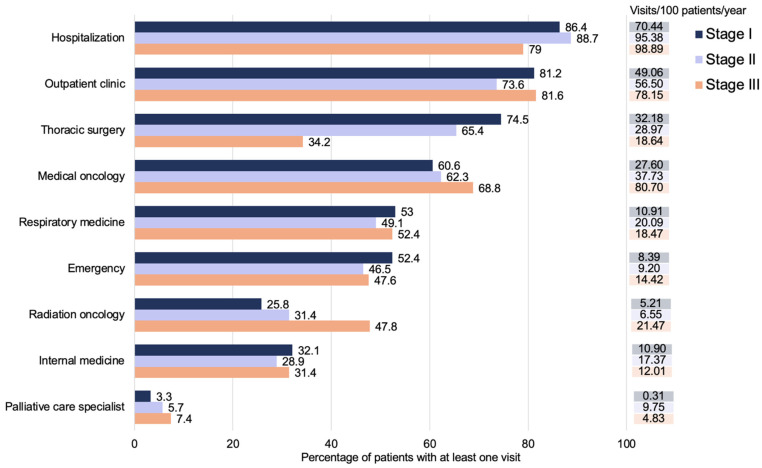
Hospitalization and hospital department visits by patients with NSCLC by disease stage. Colored bars show the percentage of patients hospitalized or with visits to hospital departments. Numbers in the right-hand column show usage rates representing the number of visits per 100 patients per person-year of follow-up.

Most patients in the study population were hospitalized at least once during the study period, including 86.4% of patients with stage I NSCLC, 88.7% with stage II, and 79% with stage III disease (Figure 3). Aside from hospitalization, the outpatient clinic was the resource used by the highest percentage of patients in all three disease stage groups (I: 81.2%, II: 73.6% and III: 81.6%), while palliative care was used by the lowest percentage of patients (I: 3.3%, II: 5.7% and III: 7.4%) (Figure 3). Usage rates of healthcare resources varied by treatment group and disease stage (Tables S6). For example, among stage I patients, usage rates for all departments except thoracic surgery (visits/100 patients/year) were numerically higher for those who received multimodal treatment with surgery, chemotherapy, and radiotherapy compared with those who received surgery alone. For patients with stage II NSCLC, usage rates for hospitalization, internal medicine, and palliative care were numerically higher in the surgery-alone group than in the groups that received combined treatment. Among stage III patients, hospitalization, medical oncology, and thoracic surgery usage rates were numerically higher in patients treated with surgery alone than in those who received chemotherapy alone.

Regarding imaging tests, computed tomography (CT) was performed on most patients in the study population (n = 897, 94.3%), as was positron emission tomography (PET, n = 788, 82.9%). Magnetic resonance imaging (MRI) and low-dose CT screening were less used (Table 4). The most common diagnostic procedure performed was biopsy, which was recorded for 716 patients (75.3%), followed by fibrobronchoscopy, recorded for 298 (31.3%) and endobronchial ultrasound (EBUS, 22.2%). There were no notable trends in imaging tests or diagnostic procedures performed when stratified by disease stage (Table 4).

## 4. Discussion

This study analyzed real-world data extracted from EHRs of adult patients diagnosed with early or locally advanced NSCLC between January 2014 and December 2018, who were followed until December 2021, using NLP- and ML-based methods. It provides a historical benchmark of clinical practice before perioperative immunotherapy and targeted therapies became available in earlier-stage disease management, capturing a pivotal period characterized by heterogeneous treatment approaches and illustrating how management strategies have changed in subsequent years. In contrast to current clinical practice, where treatment selection is increasingly guided by biomarker status and involves multimodal and personalized approaches, the patterns observed here reflect a more limited therapeutic landscape. These findings also highlight the increasing range of therapeutic options now available, and the consequent need for multidisciplinary coordination in decision-making.

The stage distribution of the study population was broadly consistent with the Spanish Thoracic Tumors Registry [27] and reflects the typical presentation of early and locally advanced NSCLC, which is most often detected in stage III when prognosis is worse compared with stage I and II disease [4,27]. These findings reinforce the value of diagnosing NSCLC at stages amenable to curative treatment [3]. During the study period, lung cancer screening had not yet been implemented in Spain, and challenges still remain for its implementation [28]. Current evidence suggests that screening criteria such as those of the United States Preventive Services Task Force (USPSTF) [29] may fail to identify more than a third of lung cancer cases in Spain, including over half of cases in women [30], highlighting the need for country-specific eligibility criteria incorporating factors such as sex, age, and smoking habit [30]. In our study population, patients were predominantly older adults with a high prevalence of current or former smoking, and a substantial proportion of women. The increasing incidence of lung cancer in women in Spain over the past two decades has been attributed to the historical peaks in tobacco smoking that occurred later in women than in men [1,31]. Nevertheless, among never-smokers, women are more frequently affected than men [27]. The substantial percentage of missing smoking data in our study highlights incomplete clinical documentation and underscores the need for improved capture of detailed information for refining future screening criteria, particularly given the rising incidence in women and never-smokers [1,32].

Treatment approaches were heterogenous across disease stages, likely reflecting differences in care practices between centers, patient or physician preferences, and the complex nature of the disease, with patient-specific factors such as comorbidities, fitness or frailty influencing clinical decisions. Given the descriptive nature of this study, guideline adherence or the clinical reasoning underlying treatment selection could not be assessed; thus, observed variability likely reflects real-world practice patterns rather than deviations from standards. Overall, a substantial proportion of patients did not undergo thoracic surgery, while surgery remained the main treatment approach in early-stage disease group. In stage III disease, surgical management was less frequent, reflecting the heterogeneous composition of resectable and unresectable tumors. These findings are consistent with the 2015 Spanish Society of Medical Oncology (SEOM) clinical practice guidelines for the management of NSCLC [33]. They are also in line with population-based data from the Netherlands covering 2008–2018, where surgery remained the most commonly delivered treatment for stage II NSCLC and a major treatment modality for stage I disease, despite evolving practice patterns over time, while multimodal and non-surgical strategies were more commonly used for stage III disease [34]. In our cohort, adjuvant treatment was commonly used, with chemoradiotherapy being the most frequent modality. Neoadjuvant therapy rates should be interpreted with caution, particularly in patients classified as stage I or II. Because pathological TNM was prioritized when available, some patients may have been classified according to post-treatment pathological stage rather than the initial clinical stage on which treatment decisions were based. Thus, neoadjuvant therapy in stage I–II may in part reflect downstaging and should not be overinterpreted as evidence of treatment selection in patients initially diagnosed with early-stage disease.

Among non-surgical patients, approximately half received chemoradiotherapy, mostly administered concomitantly, in line with contemporary guidelines for unresectable stage III disease [33], although in our study it was also used in selected stage I-II cases. A substantial proportion had no active treatment recorded. This likely reflects a heterogeneous group, including patients receiving best supportive care due to comorbidities or poor performance status, as well as cases of treatment delivered outside participating institutions or not fully captured in the EHR. This is particularly relevant in stage III disease, where not all patients are eligible for standard concurrent chemoradiotherapy. These factors should be considered when interpreting treatment patterns and associated healthcare resource use. Although early-stage NSCLC is generally resectable, a subset of stage I and II patients may not be eligible for surgery due to comorbidities or advanced age. At the time of the study, stereotactic radiotherapy was recommended for such patients [33]; however, radiotherapy alone was detected in only 21.1% of stage I and in 17.5% of stage II, with no information collected on the radiotherapy technique used. Since then, substantial advances in both surgical and radiotherapy techniques have improved patient outcomes [18,35,36,37]. Minimally invasive and robotic-assisted surgery, together with improved perioperative care, have reduced morbidity and expanded surgical eligibility [18]. Another critical change in surgical strategy since our study has been the introduction of neoadjuvant and perioperative chemoimmunotherapy. In clinical trials, neoadjuvant chemoimmunotherapy is associated with high rates of definitive surgery (80–85% of patients), with R0 resection in most cases [9], and without adversely affecting surgical feasibility or outcomes [11,13,14]. In neoadjuvant and perioperative approaches, immunotherapy has improved event-free survival, with regimens such as neoadjuvant nivolumab plus chemotherapy, and perioperative pembrolizumab demonstrating significant overall survival benefit [10,12,13,14,15]. Although PD-L1 expression has been associated with varying benefit from immunotherapy, perioperative pembrolizumab is currently the only approved regimen irrespective of PD-L1 status [9,12,13,14]. Ongoing optimization of treatment sequencing [18], together with multidisciplinary tumor board evaluation, will be essential to improve outcomes and guide surgical eligibility, treatment decisions, and coordinated care across specialties [38,39,40].

During the study period, the absence of immunotherapy and targeted therapies for NSCLC was apparent in the low rates of biomarker testing. Among the biomarkers detected, only EGFR, ALK, and PD-L1 were tested in more than 10% of patients, consistent with the emerging role of EGFR, ALK, and PD-L1 inhibitors in advanced disease. These findings should be interpreted cautiously, as anatomical pathology records were available only for a subset of participating sites, and significantly influenced biomarker detection rates. Therefore, observed testing frequencies may partly reflect under-detection related to data availability rather than true clinical practice. Notably, sensitivity analyses excluding patients from sites without pathology data yielded similar results, suggesting limited overall impact. These low testing rates are consistent with the SEOM recommendations in force during the study period (2015) [33], when molecular testing in early-stage disease was not routinely recommended and was primarily restricted to advanced stages. These guidelines were subsequently updated in 2019 [41] and 2022 [42], but continued to focus mainly on molecular testing in advanced-stage disease, with only limited consideration of its use in earlier-stage settings. In this regard, contemporary European guidelines similarly restricted molecular testing mainly to advanced-stage disease during the study period [43]. As immunotherapy and targeted therapies are increasingly used in earlier stages, early and comprehensive molecular testing becomes more relevant to guide treatment decisions. Despite this, biomarker testing in Spain and elsewhere remains challenging and underused [44,45], and current SEOM-SEAP clinical guidelines focus primarily on biomarker testing in stage IV disease [42]. These findings support consideration for extending recommendations to earlier stages and strengthened efforts to ensure adequate tissue sampling for optimal testing.

Healthcare resource use in our study was substantial and was consistent with stage distribution and treatment intensity, particularly the higher use of surgery and oncology services in early-stage disease. These findings align with previous Spanish studies identifying surgery, diagnosis, and chemotherapy as major cost drivers in NSCLC [46,47]. As treatment becomes increasingly personalized, healthcare systems will require coordinated planning to support advanced diagnostics, multimodal care, and long-term survivorship needs [38,48,49,50].

The main strength of this study is the application of NLP and ML methods to a large real-world dataset (951 patients from over 100 million EHRs), enabling comprehensive characterization of NSCLC care. Rigorous validation procedures and multisource data integration enhanced data robustness and allowed for the capture of multidisciplinary care. Nevertheless, several limitations should be acknowledged. First, the study relies on routinely recorded EHR data, which depends on documentation quality and NLP performance. Disease stage was extracted from EHRs based on explicit mentions of stage (I, II, III) near the index date or converted from TNM values within a defined time window. A dedicated NLP model identified the TNM edition when explicitly reported. Otherwise, the 8th edition was assumed for index dates from 2017 onwards, corresponding to its implementation across participating hospitals. Staging was derived using predefined prioritization rules, with pathological stage prioritized when available. Consequently, the reported stage may in some cases reflect post-treatment pathological staging rather than the initial clinical stage on which treatment decisions were based, particularly after neoadjuvant therapy, and should be considered when interpreting treatment patterns. In addition, treatment variables were reconstructed from unstructured clinical text using NLP-based rules, and surgical procedures may have been incompletely captured if performed outside participating hospitals or not recorded in the available data sources. These factors may explain apparent inconsistencies, such as neoadjuvant or adjuvant therapy in stage I disease or neoadjuvant therapy without recorded surgery. Likewise, the absence of recorded treatment does not necessarily indicate absence of treatment in clinical practice and may reflect incomplete data capture, NLP limitations, or appropriate clinical decision-making. In addition, NLP performance varied across treatment-related concepts. The lowest F1-score was observed for “stereotactic radiotherapy”, mainly due to a site-specific acronym not captured in the lexical variants. However, this reflects variability at the level of a specific treatment subtype and does not correspond to the performance of the broader radiotherapy variables, which were derived using multiple complementary criteria. Therefore, although some degree of residual misclassification cannot be excluded, the impact on the main treatment group classification is expected to be limited. Second, data availability was heterogenous across sites, as radiology, pathology, and oncology pharmacy records were only available from three of the four sites. Third, certain variables, including detailed radiotherapy protocols, chemotherapy cycles, and granular healthcare resource use by treatment approach, were insufficiently captured for robust analysis. Fourth, healthcare resource use was estimated using the number of records available per department, assuming one record per visit; consequently, visit counts may be overestimated when multiple records were generated during a single clinical encounter; however, it is unlikely to affect comparisons between study groups. Fifth, institutional differences in clinical practices, resource availability, and documentation may have contributed to variability in treatment patterns and healthcare resource use. These factors may also partly explain the moderate inter-annotator agreement (IAA) for the cohort-defining variables NSCLC and stage (0.66 and 0.77, respectively), reflecting the inherent complexity of annotating real-world clinical records, where interpretation of concept mentions may vary depending on whether external annotators based their judgement on clinical (rather than purely terminological) criteria. Finally, death ascertainment relied on hospital EHRs and did not capture out-of-hospital deaths, potentially leading to underestimation of overall mortality. Consequently, survival analyses were not included in the present study. Despite these limitations, several methodological measures, including predefined variable construction rules, expert validation, and sensitivity analyses, were implemented to minimize potential biases, supporting cautious interpretation of the findings within the context of real-world clinical practice.

## 5. Conclusions

This study used NLP and ML to analyze real-word data from the EHRs of patients with early and locally advanced NSCLC diagnosed between 2014 and 2018, who were followed until 2021. Our results present a historical snapshot of NSCLC care during a period immediately before integration of immunotherapy and targeted therapy into routine clinical practice for these patients, thus providing insight into how care strategies have evolved and will continue to change in the future. Nearly half of the patients in our study were diagnosed with stage III disease, less than 60% of the study population underwent surgery, biomarkers were largely untested, and most patients were hospitalized at least once. As we move to an era of advanced therapies, our results support care approaches focused on early diagnosis and multidisciplinary collaboration to expand surgical eligibility and determine optimal treatment strategies. In addition, structured patient pathways and coordinated resource planning will be essential for implementing complex workflows and personalized treatment plans to meet the needs of contemporary NSCLC patients and survivors.

## Figures and Tables

**Table 1:** Demographics and tumor histological characteristics of patients with NSCLC.

	Stage I n = 330	Stage II n = 159	Stage III n = 462	Total N = 951
Age, median (Q1, Q3)	67 (59, 73)	66 (57, 73)	64 (56, 71)	66 (58, 72)
Females, n (%)	112 (33.9)	50 (31.4)	141 (30.5)	303 (31.9)
Smoking habit, n (%)				
Former	52 (15.8)	29 (18.2)	53 (11.5)	134 (14.1)
Current	165 (50.0)	68 (42.8)	266 (57.6)	499 (52.5)
Never	62 (18.8)	28 (17.6)	61 (13.2)	151 (15.9)
Missing	51 (15.5)	34 (21.4)	82 (17.7)	167 (17.6)
Tumor histology, n (%) ^§^				
Adenocarcinoma	213 (64.5)	83 (52.2)	252 (54.5)	548 (57.6)
Squamous carcinoma	67 (20.3)	54 (34.0)	144 (31.2)	265 (27.9)
Missing	47 (14.2)	21 (13.2)	57 (12.3)	125 (13.1)
Comorbidities, n (%) *				
Hypertension	178 (53.9)	79 (49.7)	227 (49.1)	484 (50.9)
Chronic obstructive pulmonary disease	152 (46.1)	70 (44.0)	184 (39.8)	406 (42.7)
Dyslipidemia	129 (39.1)	59 (37.1)	148 (32.0)	336 (35.3)
Diabetes	74 (22.4)	29 (18.2)	107 (23.2)	210 (22.1)
Emphysema	43 (13.0)	21 (13.2)	66 (14.3)	130 (13.7)
Ischemic cardiopathy	45 (13.6)	16 (10.1)	52 (11.3)	113 (11.9)
Hypothyroidism	38 (11.5)	18 (11.3)	53 (11.5)	109 (11.5)
Chronic bronchitis	35 (10.6)	16 (10.1)	55 (11.9)	106 (11.1)

Variable values are captured at [First report, End of follow-up], keeping the closest value to index date, for demographic and histological characteristics, and at [First report, Index + 1 year] for comorbidities. ^§^ Variables with a frequency of less than 5% across all stages and in the overall total are not shown in the table. These include the tumor histologies large cell carcinoma and adenosquamous carcinoma. * Comorbidities shown are those that were detected in >10% of the study population.

**Table 2:** Characteristics and clinical management of patients with NSCLC who underwent surgery.

	Stage I n = 259	Stage II n = 119	Stage III n = 164	Total N = 542
Age, median (Q1, Q3)	66 (58, 71)	66 (56, 72)	63 (56, 69)	65 (57, 71)
Females, n (%)	88 (34.0)	40 (33.6)	48 (29.3)	176 (32.5)
Histology, n (%) ^§^				
Adenocarcinoma	186 (71.8)	71 (59.7)	105 (64.0)	362 (66.8)
Squamous carcinoma	52 (20.1)	40 (33.6)	51 (31.1)	143 (26.4)
Missing	18 (6.9)	7 (5.9)	5 (3.0)	30 (5.5)
Surgery type, n (%) ^§^				
Lobectomy	205 (79.2)	89 (74.8)	104 (63.4)	398 (73.4)
Pneumonectomy	15 (5.8)	18 (15.1)	31 (18.9)	64 (11.8)
Wedge resection	14 (5.4)	2 (1.7)	4 (2.4)	20 (3.7)
Unknown	15 (5.8)	8 (6.7)	22 (13.4)	45 (8.3)
Treatment type, n (%) ^§^				
Surgery alone	117 (45.2)	35 (29.4)	23 (14.0)	175 (32.3)
Adjuvant treatment	125 (48.3)	83 (69.7)	131 (79.9)	339 (62.5)
CHT + RDT	44 (17.0)	33 (27.7)	98 (59.8)	175 (32.3)
CHT	37 (14.3)	33 (27.7)	17 (10.4)	87 (16.1)
RDT	26 (10.0)	10 (8.4)	11 (6.7)	47 (8.7)
Unknown *	18 (6.9)	7 (5.9)	5 (3.0)	30 (5.6)
Neoadjuvant treatment	39 (15.1)	16 (13.4)	58 (35.4)	113 (20.8)
CHT + RDT	4 (1.5)	1 (0.8)	12 (7.3)	17 (3.0)
CHT	8 (3.1)	3 (2.5)	11 (6.7)	22 (4.1)
Unknown *	18 (6.9)	8 (6.7)	30 (18.3)	56 (10.3)
Adjuvant and neoadjuvant treatment	22 (8.5)	15 (12.6)	48 (29.3)	85 (15.7)

Variable values are captured at [First report, End of follow-up] for age, sex, and histology; at [Surgery date, Progression] for adjuvant treatments; and at [Index, Surgery date] for neoadjuvant treatments. ^§^ Variables with a frequency of less than 5% across all stages and in the overall total are not shown in the table. These include the histologies large cell carcinoma and adenosquamous carcinoma, the surgery type segmentectomy, and the neoadjuvant treatment type RDT. * Only mention of ‘adjuvant’ or ‘neoadjuvant’ was detected in free text. CHT, chemotherapy; RDT, radiotherapy; CHT + RDT, chemoradiotherapy. Treatment categories are not mutually exclusive. Patients who received both neoadjuvant and adjuvant therapy are included in both the “Neoadjuvant treatment” and “Adjuvant treatment” rows and are also reported separately under “Adjuvant and neoadjuvant treatment”.

**Table 3:** Characteristics and clinical management of patients with NSCLC who did not undergo surgery.

	Stage I n = 71	Stage II n = 40	Stage III n = 298	Total N = 409
Age, median (Q1, Q3)	71 (65, 79)	70 (60, 77)	65 (58, 72)	67 (59, 74)
Female, n (%)	24 (33.8)	10 (25.0)	93 (31.2)	127 (31.1)
Histology, n (%) ^§^				
Adenocarcinoma	27 (38.0)	12 (30.0)	147 (49.3)	186 (45.5)
Squamous carcinoma	15 (21.1)	14 (35.0)	93 (31.2)	122 (29.8)
Missing	29 (40.8)	14 (35.0)	52 (17.4)	95 (23.2)
Type of treatment, n (%)				
CHT + RDT	12 (16.9)	19 (47.5)	175 (58.7)	206 (50.4)
Concomitant	6 (50.0)	11 (57.9)	130 (74.3)	147 (71.4)
Sequential	3 (25.0)	5 (26.3)	43 (24.6)	51 (24.8)
Unknown	3 (25.0)	3 (15.8)	2 (1.1)	8 (3.9)
CHT alone	8 (11.3)	1 (2.5)	31 (10.4)	40 (9.8)
RDT alone	15 (21.1)	7 (17.5)	23 (7.7)	45 (11.0)
Total CHT (CHT + RDT & CHT)	20 (28.2)	20 (50.0)	206 (69.1)	246 (60.1)
Total RDT (CHT + RDT & RDT)	27 (38.0)	26 (65.0)	198 (66.4)	251 (61.4)
No treatment detected	36 (50.7)	13 (32.5)	69 (23.2)	118 (28.9)

Variable values are captured at [First report, End of follow-up]. Twenty-six patients received neoadjuvant treatment (defined as the mention of neoadjuvant variable) and showed no evidence of having undergone surgery. Therefore, they were classified as patients who did not undergo surgery. ^§^ Variables with a frequency of less than 5% across all stages and in the overall total are not shown in the table. These include the histologies large cell carcinoma and adenosquamous carcinoma. CHT, chemotherapy; RDT, radiotherapy; CHT + RDT, chemoradiotherapy.

**Table 4:** Imaging tests and diagnostic procedures in patients with early or locally advanced NSCLC.

	Stage I n = 330	Stage II n = 159	Stage III n = 462	Total N = 951
Imaging test, n (%) ^§^				
Computed tomography (CT)	313 (94.8)	146 (91.8)	438 (94.8)	897 (94.3)
Positron emission tomography (PET)	273 (82.7)	136 (85.5)	379 (82.0)	788 (82.9)
Magnetic resonance imaging (MRI)	145 (43.9)	52 (32.7)	199 (43.1)	396 (41.6)
Diagnostic procedure, n (%) ^§^				
Biopsy	234 (70.9)	120 (75.5)	362 (78.4)	716 (75.3)
Fibrobronchoscopy	87 (26.4)	48 (30.2)	163 (35.3)	298 (31.3)
Endobronchial ultrasound (EBUS)	45 (13.6)	33 (20.8)	133 (28.8)	211 (22.2)
Mediastinoscopy	9 (2.7)	9 (5.7)	30 (6.5)	48 (5.0)

Variable values are captured at [Index − 4 months, End of follow-up]. Percentages refer to the proportion of patients undergoing each specific and pre-specified procedure within each stage. As patients may have undergone more than one procedure, and additional diagnostic tests may have been performed but were not included, percentages are not mutually exclusive and do not add up to 100%. ^§^ Variables with a frequency of less than 5% across all stages and in the overall total are not shown in the table. These include the imaging test low-dose CT screening and the diagnostic procedures CT-guided biopsy and endoscopic ultrasound (EUS).

## Data Availability

The original contributions presented in the study are included in the article and Supplementary Material. Further inquiries can be directed to the corresponding author.

## Supplementary Materials

The following supporting information can be downloaded at https://www.mdpi.com/article/10.3390/life16071119/s1, Table S1: Evaluation of EHRead^®^ performance for identifying mentions of key variables; Table S2: General NLP models used by EHRead^®^; Table S3: Specific NLP models developed to address study objectives; Table S4: Biomarker profiles by disease stage for patients in the whole study population and in those from the three hospitals that provided anatomical pathology records for the study; Table S5. Impact of staging reassignment on neoadjuvant therapy rates in surgical patients; Table S6: Hospital department usage rates for the study population by treatment group and disease stage.

## Abbreviations

The following abbreviations are used in this manuscript:

ALK	Anaplastic lymphoma kinase
BRAF	B-RAF proto-oncogene
EHR	Electronic health record
EGFR	Epidermal growth factor receptor
KRAS	Kirsten rat sarcoma viral oncogene homolog
MET	Mesenchymal–epithelial transition factor
ML	Machine learning
NLP	Natural language processing
NSCLC	Non-small cell lung cancer
NTRK	Neurotrophic tyrosine receptor kinase
PD-1	Programmed cell death protein 1
PD-L1	Programmed death-ligand 1
RET	Rearranged during transfection
ROS-1	c-Ros oncogene-1
SNOMED CT	Systematized nomenclature of medicine clinical terms
