# Supplementary material for: Real-World Insights into Stage I–III Non-Small Cell Lung Cancer in Spain in the Pre-Immunotherapy Era Using AI Techniques: The IntellyLUNG Study
Source: Life (Basel). 2026 Jul 5;16(7):1119. doi: 10.3390/life16071119 (PMC13413279; doi:10.3390/life16071119)
Supplement: Supplementary file 1 [file life-16-01119-s001.zip › life-4355000-supplementary.pdf]

Real-World Insights into Stage I–III Non-Small Cell Lung Cancer in Spain in the Pre-immunotherapy Era using AI Techniques: The IntellyLUNG Study

Supplemental Materials

TABLE OF CONTENTS

S1. SUPPLEMENTAL METHODS 2

S1.1. Data source 2

S1.2. Data processing 2

S1.2.1. Data acquisition 2

S1.2.2. Data integration 2

S1.3. Data operations to construct relevant variables and filters for the study 2

S1.4. EHRead® Performance 4

S1.5. Sensitive analysis of anatomic pathology data 5

S2. SUPPLEMENTAL RESULTS 7

SUPPLEMENTAL TABLES

Table S1. Evaluation of EHRead® performance for identifying mentions of key variables. 8

Table S2. General NLP models used by EHRead®. 9

Table S3. Specific NLP models developed to address study objectives. 10

Table S4. Biomarker profiles by disease stage for patients in the whole study population and in those from the three hospitals that provided anatomical pathology records for the study. 11

Table S5. Impact of staging reassignment on neoadjuvant therapy rates in surgical patients. 14

Table S6. Hospital department usage rates for the study population by treatment group and disease stage. 15

S1. SUPPLEMENTAL METHODS

S1.1. Data source

Data from patients included in the study were unstructured free-text in EHRs, from four hospitals within the Spanish Healthcare Network, including admission and discharge notes, outpatient clinic notes, medical reports, emergency notes, procedure notes, and notes from all available services and departments in each participating site. The information received from different data sources was refined during different stages of the data integration process until it was integrated into a secure ecosystem. At each stage, we had intermediate information that allowed the system to determine data status, getting metrics on quality and completeness to ensure the eligibility of the participating centers. Images, such as hand-drawn pictures and scanned images, were not extracted. No data entry by physicians or their delegates into an electronic data capture platform was performed. No Clinical Research Documents were collected for this study.

S1.2. Data processing

S1.2.1. Data acquisition

The data acquisition was responsibility of the participating sites. Specific documentation listing the necessary data sources to construct the study database was created based on the study protocol. Briefly, the list of data sources contained information about the necessary type of data, hospital departments, and hospital areas needed to assess the objectives of the study. Data acquisition was done once per hospital and after the conclusion of the study period and was conducted exclusively in a retrospective manner, with no additional data extractions performed.

S1.2.2. Data integration

Medsavana received the EHRs from heterogeneous sources, as every site may have had different Information Systems. This information was then uploaded to a secure file transfer protocol utility exclusively available for each site. In the integration stage, the EHRs were included in an inventory to be prepared for the NLP phase (EHRead® technology). This step comprised format standardization, data cleaning, data quality reporting, and application of business rules.

S1.3. Data operations to construct relevant variables and filters for the study

Patient selection*:* patients were included in the study if they were diagnosed during the diagnosis period, the earliest mention of lung cancer and/or [Stage]/[TNM] was detected within the diagnosis period and there was no record of any specific lung cancer surgery, cancer treatment (including, e.g., generic "chemotherapy" or "radiotherapy" mentions), or metastasis before the start of the study period.

Lung cancer staging: disease stage was captured from the output of two dedicated NLP models (see Supplementary Table S2). When more than one value for disease stage was found, the following rules were applied sequentially:

1. If there was a gap of more than 3 months between two [Stage]/[TNM] values, the one closest to the earliest NSCLC mention was taken.
2. For variables within the same 3-month window, values were prioritized as follows: specific stage (e.g., IIA) > pathological TNM (pTNM) > clinical TNM (cTNM) > incomplete/ambiguous pTNM/cTNM (e.g., T3N1, TxN2M0, etc.).

The decision to prioritize pTNM over cTNM reflected the higher reliability of pathological staging in clinical documentation, whereas cTNM is often reported in oncology/radiology/pneumonology reports with less precision in routine practice (e.g., “T2-3” or “N1-2”).

Surgery definition: Surgery was defined as the presence of any of the following conditions:

Detection of the variables related with specific lung cancer surgery ("lobectomía de pulmón", "neumonectomía total", "resección en cuña", "resección de segmento de pulmón")

Detection of [adjuvant] from the treatment intent model, [R1/R2/R0] from the resection model or of a pathological [TNM] (pTNM or ypTNM) from the TNM model (see Supplemental Table 2).

Adjuvant and Neoadjuvant therapies: the attributes [adjuvant] or [neoadjuvant] were assigned to one or more treatments based on the output of the treatment intent model or based on their temporal detection with respect to the date of surgery (see Supplemental Table 2).

Treatment definitions: Some treatments required construction through the combination of variables:

- Radiotherapy: detection of radiotherapy variables ("radioncología Y/O radioterapia", "radioterapia de intensidad modulada", "Radioterapia 3D", "radioterapia estereotáctica", "arcoterapia volumétrica modulada") or detection of the attribute [radical] from the treatment intent model or a plausible “Grays” value (between 20 and 70).
- Chemoradiotherapy: direct detection of the term “Quimiorradioterapia”, the treatment-intent model attributes [Concomitante] or [Secuencial], or the co-occurrence of radiotherapy and chemotherapy. Concurrent chemoradiotherapy was defined as chemotherapy and radiotherapy documented within the same clinical report, whereas sequential chemoradiotherapy was defined as chemotherapy and radiotherapy recorded in separate clinical reports. Chemoradiotherapy was treated as a standalone, exclusive analysis group, so that a patient assigned to this group was excluded from the chemotherapy or radiotherapy groups.

Treatment combinations: Chemotherapy terms were individually detected in free text, and treatment combinations were identified by applying the following rules:

1. Two chemotherapy treatments were considered a combination when separated by a maximum of 120 days (4 months).
2. Nonspecific terms such as "Quimioterapia" were not considered for combinations.
3. Only the first combination detected per patient was considered, except for patients with adjuvant and neoadjuvant treatments that may have had two different combinations at different timepoints (before and after the surgery).
4. If two platinum-based drugs (Cisplatin or Carboplatin) were detected for the same combination, the one that appeared first in a report was selected.
5. When three treatments were detected within the 4-month window, only the first two treatments mentioned were considered for the combination.

PDL1 expression result: For the variable associated with the biomarker PDL-1, two NLP models were available (see Supplementary Table 2): the alteration status model, with [positive/negative] as output and the measurable parameters model, which detects numerical results. When obtaining final PDL-1 results, numeric values took precedence over categorical values. Therefore, a numeric value ≥ 1% was considered "positive". Conversely, a numeric value <1% was considered "negative".

S1.4. EHRead® Performance

The ability of EHRead® to accurately extract key study variables was evaluated using the methods published in Canales L. et al. *JMIR Med Inform*, 2021, 9(7): p.e20492. This external validation was conducted by comparing EHRead®'s detection capabilities with a reference set of annotated medical records curated by expert physicians at each participating hospital.

The performance of *EHRead®* (Supplemental Table 3) was evaluated at the clinical term level and involved the following phases:

- *Text collection*. In NLP systems, the amount of data necessary to capture enough linguistic events to ensure consistent and robust performance metrics is an open question. To address this, we utilized the Sample Calculator for Evaluation (SLiCE®), a software tool specifically developed for this purpose. This calculator indicates the minimum number of annotated EHRs required to obtain the expected parameters based on the prevalence in the EHRs of the main study variable. The parameters used for this calculation included a confidence level of 95% (α = 5%), interval widths of 10% (percentage points) and expected values of precision and recall. Thus, SliCE provides a robust estimation of precision and recall, assuring that the true value is at ±5% (percentage points) with a confidence level of 95%.

- *Annotation task*. The overall goal of this phase was to evaluate the system's accuracy when identifying records that contained mentions of NSCLC-related variables. To build the standard corpus, a set of documents was first pre-annotated using *EHRead®* technology; these documents included key study variables to identify the population with NSCLC. Then, these documents were corrected manually via an in-house-developed Evaluation Tool.
- *Annotation of the standard*. Two designated expert physicians (hereby referred to as 'the annotators') at each hospital annotated the set of randomly selected records. Annotators adhered to the annotation guidelines developed by the medical team of NLP experts. Then, the inter-annotator agreement (IAA) was measured using the F1-Score to ensure the consistency of the guidelines and the reliability of the annotation. The IAA is a metric that indicates the extent to which the different annotators converged in their evaluation, thus providing information regarding the difficulty of the task. Finally, a third physician acted as judge, reviewing the annotations made by the two annotators and resolving any possible discrepancies. The resulting standard corpus served as a resource for the evaluation of the performance of EHRead® technology.
- *Evaluation*. The evaluation of the system was calculated in terms of the standard metrics of Precision, Recall, and their harmonic mean F1-Score.
  - *Precision* = $\frac{tp}{tp + fp}$. This parameter indicates the accuracy of the system in retrieving key clinical concepts.
  - *Recall* = $\frac{tp}{tp+fn}$. This parameter indicates the amount of information the system retrieves.
  - *F1-Score* = $\frac{2xPrecisionxRecall}{Precision+Recall}$. This parameter gives us an overall performance indicator of information retrieval.

In all cases, *tp* is the number of true positives (i.e., records correctly retrieved), *fn* is the set of false negatives (i.e., records incorrectly not retrieved), and *fp* is the number of false positives (i.e., records incorrectly retrieved).

S1.5. Sensitive analysis of anatomic pathology data

Uniform data requests were sent to all study sites, but records from anatomic pathology (AP) were provided by only 3 of the 4 sites. Since this department is the main source of biomarker data, we conducted a comparative analysis to determine whether biomarker results presented in the study were impacted by the missing AP records. The analysis was performed on the proportion of patients with or without ≥1 biomarker detected (regardless of the altered/not altered result) from the hospitals with AP records compared with the hospital that did not send AP records. The

analysis showed that mention of ≥1 biomarker was detected in 45% of patients in hospitals with AP records, compared with 35% for the hospital without. The difference was statistically significant (chi-square $p<0.05$), indicating that having AP records does impact biomarker detection rate. Therefore, the biomarker results were recalculated based only on patients from the 3 hospitals with AP records included in the study database (Table S4). The recalculated proportions did not reveal any changes to the study conclusions.

S2. SUPPLEMENTAL RESULTS

Among patients classified as stage I who received neoadjuvant therapy, 12 out of 39 had evidence of a higher earlier clinical stage, and, similarly, among those classified as stage II, 3 out of 16 had a prior stage III. Table S5 summarizes the potential impact of staging reassignment on neoadjuvant therapy rates. Stage-specific estimates are presented as ranges to reflect uncertainty in the redistribution of patients originally classified as stage I, whereas the combined stage II–III estimate provides a simplified aggregated scenario.

As an exploratory assessment, assuming deterministic reassignment of stage II patients and a plausible redistribution of stage I patients, neoadjuvant therapy rates would decrease from 15.1% to 10.9% in stage I. In stage II, rates would range from 11.2% to 19.5% (compared with the original 13.4%), and in stage III from 36.5% to 40.7% (compared with the original 35.4%).

When stages II and III are considered jointly to account for uncertainty in the redistribution between these stages, the overall neoadjuvant therapy rate increases from 26.1% to 29.1%. This aggregated estimate is consistent with the more detailed range-based analysis and provides additional context on the magnitude of the staging hierarchy effect.

Table S1. Evaluation of EHRead® performance for identifying mentions of key variables.

Variable	Detections	Precision	Recall	F1-score	IAA
Lung adenocarcinoma	515	0.82	0.82	0.82	0.94
Lung lobectomy	507	0.96	0.95	0.96	0.99
Stage	439	0.94	0.85	0.89	0.77
Radiation oncology and/or radiotherapy	430	0.76	0.96	0.85	0.78
Chemotherapy	310	0.75	0.91	0.82	0.91
Carboplatin	241	0.87	0.83	0.85	0.95
Pemetrexed	207	0.95	0.99	0.97	0.94
Non-small cell lung cancer	186	0.94	0.94	0.94	0.66
Cisplatin	153	0.97	0.99	0.98	0.98
Squamous cell lung carcinoma	144	0.81	0.78	0.80	0.91
Vinorelbine	126	0.98	0.99	0.98	0.96
Paclitaxel	125	0.94	0.93	0.93	0.98
Chemoradiotherapy	112	0.77	0.87	0.82	0.96
Stereotactic radiotherapy	60	0.70	0.77	0.73	0.74

Precision: ratio between true positives (annotator marks) and total positive detections (annotator marks plus NLP predictions).
Recall: ratio between true positives and true positives plus false negatives.
F1-score: harmonic mean between precision and recall, and summary metric for model performance.
IAA: inter-annotator agreement

Table S2. General NLP models used by EHRead®.

Model	Description
General named-entity recognition	Baseline detection of clinical entities (including acronyms) using a combination of both ML and rule-based entity-recognition methods.
General named-entity linking	Linking of terminology identifiers to the detected clinical entities (including acronyms) using both explicit linking via rules and lexical/semantic linking against Medsavana's terminology using ML methods.
Negation detection	Detection of negation markers and classification of the status of clinical entities into 'affirmative' or 'non-affirmative' using a combination of both ML and rule-based methods.
Section detection	Classification of EHRs' free-text paragraphs into predefined classes such as "family history", "patient present" or "patient past" using a combination of both ML and rule-based entity-recognition methods.
Temporality	Detection of datetime mentions in EHRs' free-text and subsequent relation-detection of the datetime and the clinical entity to which it refers to using a combination of both ML and rule-based methods.
Measurable parameters	Detection of laboratory test results and subsequent relation-detection of the test result and the clinical entity to which it refers to using a combination of both ML and rule-based methods.

EHR, electronic health record; ML, machine learning; NLP natural language processing.

Table S3. Specific NLP models developed to address study objectives.

Model	Clinical entities‡	Attributes§	Model output handling
Stage	Lung Cancer,	I, II, III, IV	Tumour staging (patients with stage IV were not included)
TNM	ADC, SQCC, LCC, TNM, Stage	T*N*M*	
TNM edition	TNM	7th, 8th	TNM edition
Treatment intent	Chemotherapy, Treatment, Radiotherapy, Chemoradiation	Neoadjuvant, Adjuvant, Concomitant, Sequential, Radical, Rescue, Palliative	Classify treatment patterns
Body parts	ADC, SQCC, LCC	Anatomical location	Primary tumour and Metastasis anatomical location
Tumour response	Response, Progression, Relapse, Stable Disease	Complete, Partial, Stable Disease, Relapse Progression	Disease outcomes
Exitus	Exitus, Death	Patient, Relative, Non-conclusive	Exclude non-patient deaths.
Alteration status	ALK, BRAF, EGFR, HER2, KRAS, MET, RET, ROS-1, TKI, PDL1	Altered, Not altered	Mutation status classification
Resection	adenocarcinoma of the lung, malignant lung tumor, squamous cell carcinoma, large cell carcinoma, non-small cell lung cancer, squamous cell carcinoma of the lung, large cell carcinoma of the lung, adenomatous malignancy, wedge resection, lung segment resection, lung lobectomy, total pneumonectomy	R0 (including R0 and "free margins"), R1, R2, Residual	Surgical margins classification

‡Entities detected in free text and structured data or extracted from structured data that can be linked to an identifier in the terminology. §Parts of the clinical text that further describe a clinical entity (e.g., location, severity), but that are not linkable to an identifier in the terminology by themselves.

ADC, adenocarcinoma; ALK, anaplastic lymphoma kinase; BRAF, B-RAF proto-oncogene; EGFR, epidermal growth factor receptor; HER2, human epidermal growth factor receptor type 2; KRAS, Kirsten rat sarcoma viral oncogene homolog; LCC, large cell carcinoma; MET, mesenchymal–epithelial transition factor; PDL1, programmed death ligand 1; RET, rearranged during transfection; ROS-1, c-Ros oncogene-1; SQCC, squamous cell carcinoma; TKI, tyrosine receptor kinase; TNM, tumor, nodes, metastasis.

Table S4. Biomarker profiles by disease stage for patients in the whole study population and in those from the three hospitals that provided anatomical pathology records for the study.

	Study population				Patients from hospitals with pathology records			
**Biomarker**	**Stage I n = 330**	**Stage II n = 159**	**Stage III n = 462**	**Total N = 951**	**Stage I n = 243**	**Stage II n = 124**	**Stage III n = 359**	**Total N = 726**
	n (%)	n (%)	n (%)	n (%)	n (%)	n (%)	n (%)	n (%)
Tumor Proportion Score*								
Negative (<1%)	18 (5.5)	9 (5.7)	54 (11.7)	81 (8.5)	12 (4.9)	2 (1.6)	45 (12.5)	59 (8.1)
Positive (≥1%)								
Unclassified	2 (0.6)	0 (0.0)	2 (0.4)	4 (0.4)	2 (0.8)	0 (0.0)	2 (0.6)	4 (0.6)
1-49%	9 (2.7)	7 (4.4)	17 (3.7)	33 (3.5)	6 (2.5)	6 (4.8)	14 (3.9)	26 (3.6)
>50%	11 (3.3)	3 (1.9)	23 (5.0)	37 (3.9)	10 (4.1)	2 (1.6)	18 (5.0)	30 (4.1)
Untested	290 (87.9)	140 (88.1)	366 (79.2)	796 (83.7)	213 (87.7)	114 (91.9)	280 (78.0)	607 (83.6)
EGFR								
Altered	28 (8.5)	14 (8.8)	37 (8.0)	79 (8.3)	24 (9.9)	11 (8.9)	33 (9.2)	68 (9.4)
Non-altered	73 (22.1)	29 (18.2)	135 (29.2)	237 (24.9)	64 (26.3)	23 (18.5)	116 (32.3)	203 (28.0)
Untested	229 (69.4)	116 (73(	290 (62.8)	635 (66.8)	155 (63.8)	90 (72.6)	210 (58.5)	455 (62.7)
ALK								
Altered	4 (1.2)	2 (1.3)	9 (1.9)	15 (1.6)	4 (1.6)	2 (1.6)	9 (2.5)	15 (2.1)
Non-altered	54 (16.4)	26 (16.4)	107 (23.2)	187 (19.7)	46 (18.9)	18 (14.5)	87 (24.2)	151 (20.8)
Untested	272 (82.4)	131 (82.4)	346 (74.9)	749 (78.8)	193 (79.4)	104 (83.9)	263 (73.3)	560 (77.1)
ROS-1								
Altered	1 (0.3)	0 (0.0)	1 (0.2)	2 (0.2)	1 (0.4)	0 (0.0)	0 (0.0)	1 (0.1)
Non-altered	21 (6.4)	10 (6.3)	48 (10.4)	79 (8.3)	17 (7.0)	4 (3.2)	44 (12.3)	65 (9.0)
Untested	308 (93.3)	149 (93.7)	413 (89.4)	870 (91.5)	225 (92.6)	120 (96.8)	315 (87.7)	660 (90.9)

KRAS								
Altered	11 (3.3)	4 (2.5)	15 (3.2)	30 (3.2)	9 (3.7)	3 (2.4)	13 (3.6)	25 (3.4)
Non-altered	12 (3.6)	7 (4.4)	21 (4.5)	40 (4.2)	12 (4.9)	4 (3.2)	19 (5.3)	35 (4.8)
Untested	307 (93.0)	148 (93.1)	426 (92.2)	881 (92.6)	222 (91.4)	117 (94.4)	327 (91.1)	666 (91.7)
BRAF								
Altered	3 (0.9)	1 (0.6)	4 (0.9)	8 (0.8)	3 (1.2)	1 (0.8)	1 (0.3)	5 (0.7)
Non-altered	5 (1.5)	1 (0.6)	6 (1.3)	12 (1.3)	2 (0.8)	0 (0.0)	6 (1.7)	8 (1.1)
Untested	322 (97.6)	157 (98.7)	452 (97.8)	931 (97.9)	238 (97.9)	123 (99.2)	352 (98.1)	713 (98.2)
MET								
Non-altered	2 (0.6)	1 (0.6)	2 (0.4)	5 (0.5)	1 (0.4)	1 (0.8)	0 (0.0)	2 (0.3)
Untested	328 (99.4)	158 (99.4)	460 (99.6)	946 (99.5)	242 (99.6)	123 (99.2)	359 (100.0)	724 (99.7)
RET								
Altered	1 (0.3)	0 (0.0)	0 (0.0)	1 (0.1)	1 (0.4)	0 (0.0)	0 (0.0)	1 (0.1)
Non-altered	2 (0.6)	0 (0.0)	0 (0.0)	2 (0.2)	0 (0.0)	0 (0.0)	0 (0.0)	0 (0.0)
Untested	327 (99.1)	159 (100.0)	462 (100.0)	948 (99.7)	242 (99.6)	124 (100.0)	359 (100.0)	725 (99.9)
HER2								
Altered	2 (0.6)	0 (0.0)	1 (0.2)	3 (0.3)	2 (0.8)	0 (0.0)	1 (0.3)	3 (0.4)
Non-altered	3 (0.9)	1 (0.6)	0 (0.0)	4 (0.4)	2 (0.8)	1 (0.8)	0 (0.0)	3 (0.4)
Untested	325 (98.5)	158 (99.4)	461 (99.8)	944 (99.3)	239 (98.4)	123 (99.2)	358 (99.7)	720 (99.2)
NTRK								
Non-altered	0 (0.0)	0 (0.0)	1 (0.2)	1 (0.1)	0 (0.0)	0 (0.0)	0 (0.0)	0 (0.0)
Untested	330 (100.0)	159 (100.0)	461 (99.8)	950 (99.9)	243 (100.0)	124 (100.0)	359 (100.0)	726 (100.0)

*Biomarkers are captured at [First report, Metastasis]. *The tumor proportion score (TPS) variable was derived by applying a model to classify cases as either positive or negative for 'PD-L1' (programmed death ligand-1) and from numerical values ranging from 0% to 100% detected around 'PD-L1' mentions. The 'Positive unclassified' category includes cases where PD-L1 was identified as positive but lacked an associated numerical value. 'Altered' refers to mutations, deletions, insertions, fusions, amplifications and rearrangements, as applicable in each case.*

EGFR, epidermal growth factor receptor; ALK, anaplastic lymphoma kinase; ROS-1, c-Ros oncogene-1; KRAS, Kirsten rat sarcoma viral oncogene homolog; BRAF, B-RAF proto-oncogene; MET, mesenchymal–epithelial transition factor; RET, rearranged during transfection; HER2, human epidermal growth factor receptor type 2; NTRK, neurotrophic tyrosine receptor kinase.

Table S5. Impact of staging reassignment on neoadjuvant therapy rates in surgical patients.

	Original Results (Table 2)			Exploratory reassignment		
	**Stage I n = 259**	**Stage II n = 119**	**Stage III n = 164**	**Stage I n = 247**	**Stage II n = 116 - 128**	**Stage III n = 167 - 179**
Neoadjuvant treatment	39 (15.1)	16 (13.4)	58 (35.4)	27 (10.9)	13 (11.2) – 25 (19.5)	61 (36.5) – 73 (40.7)
	**Stage I n = 259**	**Stage II–III n = 283**		**Stage I n = 247**	**Stage II–III n = 295**	
Neoadjuvant treatment	39 (15.1)	74 (26.1)		27 (10.9)	86 (29.1)	

Reassignment was based on available information on earlier clinical stage. Patients originally classified as stage II with prior stage III (n=3) were reassigned deterministically to stage III, whereas patients originally classified as stage I with a prior higher stage (n=12) were redistributed across stages II–III, generating ranges to reflect uncertainty. Denominators were adjusted accordingly for each scenario.

Table S6. Hospital department usage rates for the study population by treatment group and disease stage.

**Stage I**	**Usage rate** (visits/100 patients/year)						
**Department**	**No treatment n = 36**	**Surgery alone n = 117**	**Surgery + CHT n = 41**	**Surgery + CHT + RDT n = 50**	**Surgery + RDT n = 26**	**Surgery + Unsp n = 25**	**Total* N = 330**
Hospitalization	97.46	63.21	81.02	76.5	80.05	33.98	70.44
Outpatient clinic	54.49	35.6	47.36	68.72	60.93	37.26	49.06
Thoracic surgery	11.41	50.19	23.58	22.48	29.25	20.54	32.18
Medical oncology	44.06	14.7	24.5	48.51	19.8	17.13	27.60
Respiratory medicine	15.9	8.49	10.13	14.9	16.99	4.03	10.91
Internal medicine	30.71	2.99	7.76	28.81	8.15	2.25	10.90
Emergency	8.74	4.52	9.07	15.19	8	4.50	8.39
Radiation oncology	12.26	3.26	1.16	8.03	3.2	2.18	5.21
Palliative care	1.34	0	1.3	0.12	0.08	0.14	0.31

** The Total column includes all treatment groups. Usage rates for treatment groups with low numbers (<20 patients) are not shown in the table: CHT (n = 8), CHT + RDT (n = 12), and RDT (n = 15). Therefore, the total may not correspond to the sum of the categories shown.*

**Stage II**	**Usage rate** (visits/100 patients/year)			
**Department**	**Surgery alone n = 35**	**Surgery + CHT n = 32**	**Surgery + CHT + RDT n = 37**	**Total* N = 159**
Hospitalization	86.95	75.81	58.3	95.38
Outpatient clinic	34.1	70.52	62.62	56.50
Medical oncology	26.15	48.61	29.7	37.73
Thoracic surgery	35.42	36.47	33.36	28.97
Respiratory medicine	8.37	11.99	16.72	20.09
Internal medicine	15.55	4.85	14.15	17.37
Palliative care	0.70	0.15	0.29	9.75

Emergency	4.95	6.54	11.66	9.20
Radiation oncology	1.74	5.07	9.83	6.55

** The Total column includes all treatment groups. Usage rates for treatment groups with low numbers (<20 patients) are not shown in the table: CHT (n = 1), CHT + RDT (n = 19), No treatment (n = 13), RDT (n = 7), Surgery + Unsp (n = 7), and Surgery + RDT (n = 8). Therefore, the total may not correspond to the sum of the categories shown.*

**Stage III**	**Usage rate** (visits/100 patients/year)							
**Department**	**CHT n = 31**	**CHT + RDT n = 175**	**No treatment n = 69**	**RDT n = 23**	**Surgery alone n = 23**	**Surgery + CHT n = 23**	**Surgery + CHT + RDT n = 108**	**Total* N = 462**
Hospitalization	115.82	122.18	86.37	52.02	163.81	33.68	73.75	98.89
Medical oncology	93.23	113.43	70.35	36.04	110.02	33.44	63.33	80.7
Outpatient clinic	105.24	93.44	70.94	36.86	39.04	45.53	80.74	78.15
Radiation oncology	12.01	29.87	10.61	6.03	2.99	8.21	23.68	21.47
Thoracic surgery	6.29	1.82	5.86	10.11	39.04	22.65	25.14	18.64
Respiratory medicine	47.47	18.06	20.55	17.45	8.03	14.78	17.77	18.47
Emergency	23.16	16.17	16.55	8.64	2.99	10.56	14.36	14.42
Internal medicine	27.74	14.99	15.51	8.64	4.48	1.64	10	12.01
Palliative care	7.15	7.07	12.17	0	0	2.7	1.79	4.83

** The Total column includes all treatment groups. Usage rates for treatment groups with low numbers (<20 patients) are not shown in the table: Surgery + RDT (n = 4), and Surgery + Unsp (n = 6). Therefore, the total may not correspond to the sum of the categories shown.*

In all Stages:
Department visits are considered for the time window [Index, EoFU].
Usage rates were calculated as the sum of all observed department visits of each resource divided by the sum of all observed follow-up times, and are presented as rates per person-year of follow-up.
CHT, chemotherapy; RDT, radiotherapy; Unsp; unspecified treatment.
